# Sleep quality and renal function among Chinese incoming college freshmen: the mediating role of lifestyle behaviors

**DOI:** 10.3389/fpubh.2025.1502947

**Published:** 2025-06-06

**Authors:** Huiying Wang, Jiali Li, Yaohui Han, Shilei Zhai, Yumeng Liu, Peipu Shen, Guifang Shen, Lishun Xiao

**Affiliations:** ^1^Department of Health Management Center, The Affiliated Hospital of Xuzhou Medical University, Xuzhou, China; ^2^School of Public Health, Xuzhou Medical University, Xuzhou, China

**Keywords:** sleep quality, renal function, incoming college freshmen, biochemical indicators, China

## Abstract

**Objective:**

This study investigates the association between sleep quality and renal function indicators, with a focus on how lifestyle behaviors mediate this relationship among Chinese incoming college freshmen during post-examination vacation period.

**Methods:**

This cross-sectional study included 3,743 non-smoking, non-drinking freshmen from two universities in Xuzhou, China. Data on demographics, sleep quality, and blood biochemical indicators were collected through self-administered questionnaires, Pittsburgh Sleep Quality Index (PSQI) and physical examinations. Multiple logistic regression (MLR) was applied to explore the related biochemical indicators associated with sleep quality. Structural equation modeling (SEM) was subsequently used to evaluate the mediating effects of lifestyle factors in this relationship.

**Results:**

Higher creatinine levels (OR = 1.01, *p* = 0.002) increased the risk of poor sleep quality, while higher urea levels (OR = 0.87, *p* < 0.001) decreased it. Unhealthy lifestyle behaviors were also associated with sleep quality, including habitual caffeinated beverage intake (OR = 1.11, *p* = 0.003) and daily screen time (OR = 1.08, *p* = 0.001). Stratified analyses by gender further supported these associations, especially in females. SEM revealed that sleep quality could affect renal function (represented by creatinine and urea) though the independent mediating effect of daily screen time and the chain mediating effect of caffeinated beverage intake and daily screen time.

**Conclusion:**

These findings suggest that promoting healthy sleep, limiting screen exposure, and reducing caffeine consumption may help protect renal health in incoming college freshmen.

## 1 Introduction

Incoming college freshmen represent a distinct population undergoing substantial life transitions. Following intensive preparation for college entrance examinations, they often experience an abrupt shift from a high-stress academic environment to a relatively relaxed holiday period. Such an abrupt transition may disrupt previously established routines, contributing to irregular sleep patterns and circadian rhythm disturbances (1–3). For instance, the Freshman Sleep and Health Study reported a significant decrease in average total sleep time from approximately 7.3 h before enrollment to 6.9 h by the end of the first quarter. Sleep variability simultaneously increased, reflecting more irregular sleep schedules, which were associated with greater gains in body mass index (BMI). Moreover, excessive smartphone use is common, potentially further compromising sleep quality (4).

Accumulating evidence indicates that poor sleep quality may not only affect behavior and academic performance, but also trigger physiological changes with multiple organ systems (5). These effects are largely mediated by neuroendocrine and inflammatory pathways, such as increased sympathetic nervous system activity, impaired glucose metabolism, systemic inflammation, and elevated blood pressure, all of which may compromise renal function (6). Under normal physiological conditions, sleep regulates key hormonal pathways controlling renal function, particularly via the renin-angiotensin-aldosterone system, which demonstrates prominent circadian variation during sleep. When acute sleep deprivation occurs, plasma cortisol levels rise; over time, chronic circadian disruption leads to elevated pro-inflammatory markers such as tumor necrosis factor-alpha and C-reactive protein, thereby creating a hormonal and inflammatory milieu detrimental to renal function (7).

However, major existing studies have focused on older adults or patients with established chronic conditions, while relatively little attention has been paid to younger, ostensibly healthy populations, particularly during the post-examination vacation period, a vulnerable health window for incoming college freshmen (8, 9). Moreover, existing studies often overlook the potential mediating role of lifestyle behaviors—such as caffeine intake and smartphone overuse, which are highly prevalent and modifiable among college freshmen. Furthermore, a notable limitation of prior research is the lack of rigorous statistical validation, such as regression diagnostics, to ensure the robustness and reliability of observed associations.

Therefore, based on existing evidence, we hypothesized that sleep quality is associated with renal function indicators (urea and creatinine) among incoming college freshmen, and that this relationship may be partially explained by lifestyle-related factors such as caffeine intake and screentime. This investigation seeks to provide insights into the early renal health risks associated with poor sleep quality in young adults and to inform potential behavioral interventions.

## 2 Methods

### 2.1 Ethical approvement

This study was conducted in accordance with the Declaration of Helsinki. It was approved by the Ethics Committee of Xuzhou Medical University (XYFY2023-KL396-01). All participants provided informed consent via a mobile electronic questionnaire that included the study information; consent was required before proceeding with the survey. The confidentiality of data and personal information was guaranteed.

### 2.2 Participants and procedure

This cross-sectional study was conducted between September and October 2023 at Xuzhou Medical University and the Jiangsu Vocational Institute of Architectural Technology in Xuzhou, China. Physical examinations for incoming freshmen were performed by trained medical staff from the Health Management Center of the Affiliated Hospital of Xuzhou Medical University. During the examination period, all freshmen were invited to complete a self-administered questionnaire via the Questionnaire Star platform.^1^ The questionnaire collected information on sociodemographic characteristics, lifestyle behaviors, and incorporated the Pittsburgh Sleep Quality Index (PSQI) to assess sleep quality. Biochemical and hematological indicators were obtained from routine physical examinations conducted at the Affiliated Hospital of Xuzhou Medical University through routine blood tests and biochemical analyses.

**Figure 1:**
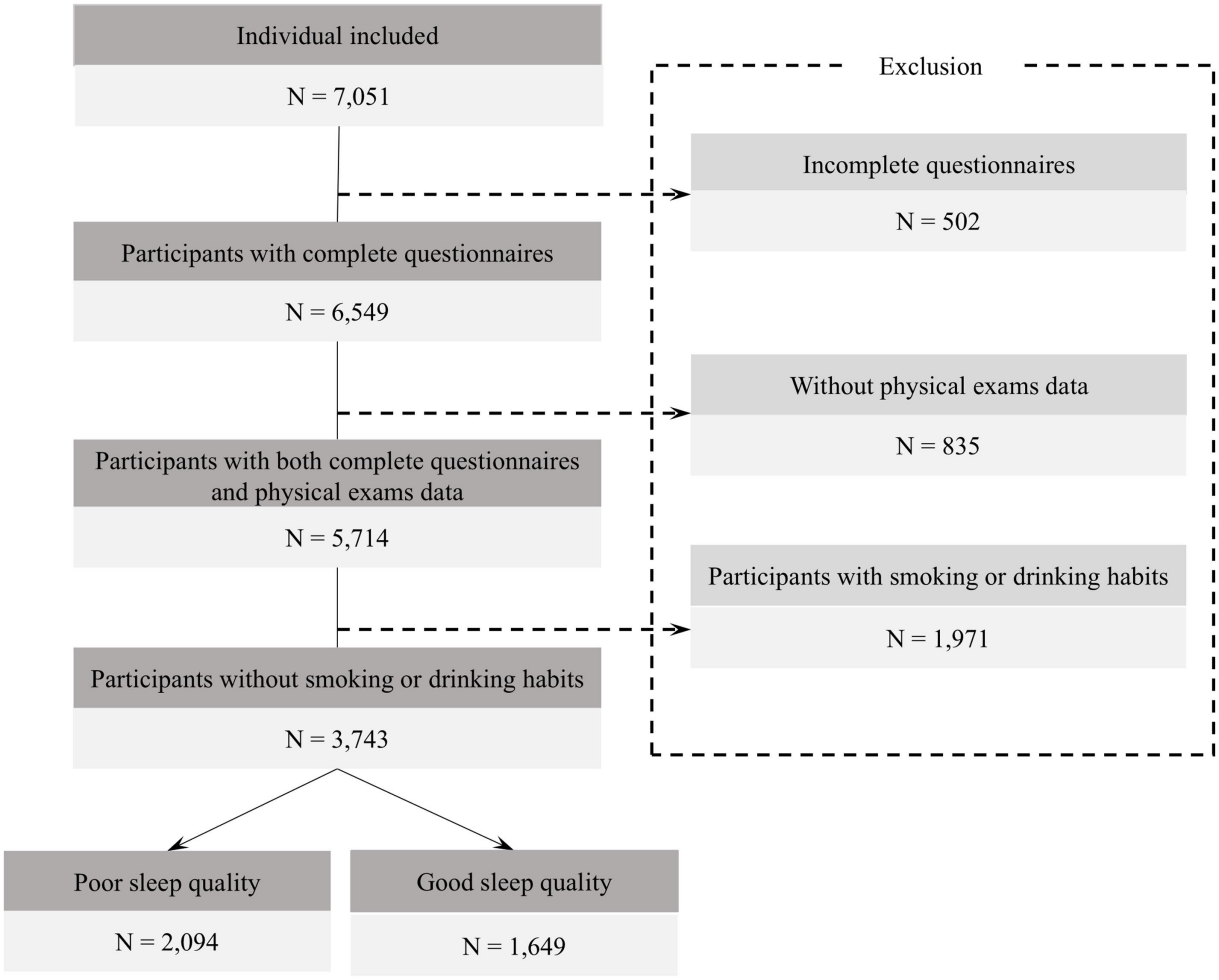
Flowchart of population included in our final analysis (*N* = 3,743).

A total of 7,051 questionnaires were collected, of which 6,549 were valid, resulting in an effective response rate of 92.88%. 2,806 participants were excluded (Figure 1). To ensure data accuracy, participants completed the questionnaire on-site during the physical examination, with guidance from trained staff. Each questionnaire was matched to physical exam data via a unique ID; unmatched or incomplete records were excluded. Logical consistency checks were also applied to improve data quality. For biochemical indicators with values below the limit of detection, imputation was conducted using the standard substitution method of limit of detection divided by the square root of two (10).

### 2.3 Measurement

#### 2.3.1 Sociodemographic variables

Participants provided information on sociodemographic characteristics including age, gender (male, female), college level (junior college, undergraduate), father’s/mother’s educational level (high school or below, junior college, undergraduate or higher), family economic status (poor, fair, good), and monthly living expenses (< 1,000, 1,000–2000, > 2000).

#### 2.3.2 Lifestyle variables

Participants reported various lifestyle behaviors over the past month. Habitual caffeinated beverage intake was categorized as never; rarely (1–2 times per month); occasionally (1–2 times per week); frequently (3–5 times per week); and very frequently (>5 times per week). Daily screen time was grouped into: 1–2 h, 2–3 h, 3–4 h, 4–5 h, 5–6 h, and 6 h or more. Primary physical activity types included leisure-related, computer game-related, travel-related, physical labor-related, mental labor-related, and others. Daily natural light exposure was reported as almost never, < 30 min, 30 min to 1 h, 1-2 h, > 2 h. Data on siesta habits were assessed by asking: “Did you have a habit of taking a siesta during the past month?” Participants who answered “no” were considered to have no siesta (0 min). Those who answered “yes” were subsequently asked to report the typical duration of each siesta episode (10 min, 15 min, 20 min, 30 min, 45 min, 60 min, 120 min, or 180 min).

#### 2.3.3 Sleep quality

The PSQI was utilized to assess the sleep quality of participants. This widely recognized tool evaluates sleep quality over the preceding month by examining seven distinct components: subjective sleep quality, sleep latency, total sleep duration, habitual sleep efficiency, sleep disturbances, use of sleep-inducing medications, and daytime dysfunction. Each component is rated on a scale from 0 to 3, yielding a composite score that ranges from 0 to 21. Higher scores on this index indicate poorer sleep quality. In this study, a PSQI cutoff of > 4 was adopted to improve sensitivity in detecting early-stage sleep disturbances among this specific population, which is acceptable in context-specific research (11).

#### 2.3.4 Clinical and laboratory measurements

The measurements were categorized into three main domains: anthropometric measurements, blood pressure, and biochemical analyses. Anthropometric measurements: Height and weight were measured by trained personnel using an ultrasonic stadiometer, with participants in light clothing, barefoot and in an upright position. BMI was calculated as weight (kg) divided by the square of height (m^2^). Blood pressure: Diastolic blood pressure (DBP), and systolic blood pressure (SBP) were measured twice using a mercury sphygmomanometer, with participants seated and having rested for at least 5 min. The two measurements were taken at an interval of no less than 2 min, and the average value was used for analysis. Biochemical analyses: Venous blood samples were collected in the morning after at least 10 h of overnight fasting. Samples were processed immediately, and serum was separated without freezing. All biochemical indicators were measured using an automated biochemical analyzer. These included hematological parameters: hematocrit, hemoglobin, red blood cells, mean corpuscular volume, mean corpuscular hemoglobin, mean corpuscular hemoglobin concentration, red cell distribution width, white blood cell count, neutrophils, lymphocytes, eosinophils, basophils, monocytes, lymphocyte percentage, eosinophil percentage, neutrophil percentage, basophil percentage, platelets, mean platelet volume, platelet distribution width (PDW); Liver function: alanine aminotransferase (ALT); Renal function: urea, creatinine, and uric acid.

### 2.4 Statistical analysis

Continuous variables were summarized using mean ± standard deviation (SD), while categorical and ordinal variables were described as frequencies and percentages. For group comparisons, the assumption of homogeneity of variances was first tested using Levene’s test. If this assumption was met, independent samples t-tests (for two groups) or one-way ANOVA (for three or more groups) were used. In cases where the assumption was violated, Welch’s t-test or ANOVA was employed accordingly. Post-hoc analyses were conducted following ANOVA, with Tukey’s test used under homogeneous variances and Games-Howell’s test under heterogeneous variances.

In regression analyses, ordinal variables were treated as continuous variables in the regression models under the assumption of a linear trend across categories. Multiple logistic regression (MLR) was employed to explore associations between sleep quality (categorized as good or poor) and blood biochemical indicators. Biochemical indicators were initially screened as independent variables, and those with variance inflation factors (VIF) greater than 10 were excluded to reduce multicollinearity. The results are presented as odds ratios (OR) and 95% confidence intervals (CI) (12). A rigorous diagnostic procedure was conducted to assess whether the MLR model satisfies key assumptions (13). These included: (1) Outlier detection and exclusion to prevent skewed results (Supplementary Figure 1). (2) Testing residual independence using the Durbin–Watson statistic; (3) Assessing the normality and homoscedasticity of residuals (Supplementary Figure 2). The model’s discrimination ability was evaluated using the receiver operating characteristic (ROC) curve, yielding an area under the curve (AUC) of 0.64 (Supplementary Figure 3). The linear trend test was performed by transforming it into a four valued ordinal variable by quartiles in the MLR model.

Among the examined biochemical indicators, only serum urea and creatinine were significantly associated with sleep quality in the MLR analysis. Based on these findings, we further explored potential pathways linking sleep quality to renal function. Given that both daily screen time and habitual caffeinated beverage intake were also significantly related to sleep quality in MLR, and are supported by previous literature as modifiable behavioral factors, they were included as potential mediators (5, 14). To simultaneously examine the direct and indirect effects of sleep quality on renal function, a structural equation model (SEM) was employed. In this model, sleep quality was measured by PSQI scores, habitual caffeinated beverage intake and daily screen time were used to quantify lifestyle factors, and renal function was represented by serum creatinine and urea levels. Sleep quality as the independent variable, caffeine intake and screen time as mediating variables, and renal function as the dependent variable. Model estimates were reported with standardized coefficients, standard errors (S. E.), and 95% CI. Bootstrapping with 2,000 iterations was performed. Model fit was evaluated using the root mean square error of approximation (RMSEA), with values ≤ 0.08 indicating an acceptable fit, and the comparative fit index (CFI), with values ≥ 0.90 suggesting a good fit of the model to the data.

All statistical analyses were conducted using R software of version 4.2.2. The “lavaan” packages were used for and SEM analyses. The significance threshold was set at *p* < 0.05.

## 3 Results

### 3.1 Baseline characteristics

Based on PSQI scores, 1,649 students (44.05%) were identified as having poor sleep quality, with a significantly higher prevalence among females compared to males (*p* < 0.001). Most participants (81.33%) were recruited from Jiangsu Province (Supplementary Table 1). Significant differences were also observed in various sociodemographic factors including parental education levels, family economic status, monthly living expenses, habitual caffeinated beverage intake, daily screen time, travel-related physical activity, mental labor-related physical activity, daily natural light exposure, and siesta duration (all *p* < 0.05). In terms of biochemical indicators, students with poor sleep quality had significantly lower levels of serum urea, red blood cells, hemoglobin, and hematocrit compared to those with good sleep quality (all *p* < 0.05), as shown in Table 1.

**Table 1:** Baseline characteristics of the study participants according to sleep quality (*N* = 3,743).

Variables	Total	Good sleep quality	Poor sleep quality	*p*
	(*N* = 3,743)	(*N* = 2094)	(*N* = 1,649)	
Age (year)	18.46 ± 0.71	18.46 ± 0.73	18.46 ± 0.67	0.998
Gender: Male	1738 (46.43)	1,028 (49.05)	713 (43.29)	**< 0.001**
Female	2005 (53.57)	1,068 (50.95)	934 (56.71)	
College level: Junior college	2,374 (63.43)	1,215 (57.97)	1,159 (70.37)	**< 0.001**
Undergraduate	1,369 (36.57)	881 (42.03)	488 (29.63)	
Father’s educational level: High school or below	2,803 (74.89)	1,530 (73.00)	1,281 (77.78)	**< 0.001**
Junior college	526 (14.05)	303 (14.46)	218 (13.24)	
College or higher	414 (11.06)	263 (12.55)	148 (8.99)	
Mother’s educational level: High school or below	2,962 (79.13)	1,612 (76.91)	1,348 (81.85)	**< 0.001**
Junior college	477 (12.74)	290 (13.84)	188 (11.41)	
College or higher	304 (8.12)	194 (9.26)	111 (6.74)	
Family economic status: Poor	484 (12.93)	210 (10.02)	274 (16.64)	**< 0.001**
Fair	3,110 (83.09)	1791 (85.45)	1,320 (80.15)	
Good	149 (3.98)	95 (4.53)	53 (3.22)	
Monthly living expense: < 1,000	633 (16.91)	284 (13.55)	349 (21.19)	**< 0.001**
1,000–2000	2,872 (76.73)	1,647 (78.58)	1,225 (74.38)	
>2000	238 (6.36)	165 (7.87)	73 (4.43)	
Habitual caffeinated beverage intake: Never	1,039 (27.76)	587 (28.01)	449 (27.26)	**< 0.001**
Rarely (1–2 times/month)	645 (17.23)	400 (19.08)	250 (15.18)	
Occasionally (1–2 times/week)	1703 (45.5)	930 (44.37)	772 (46.87)	
Frequently (3–5 times/week)	328 (8.76)	169 (8.06)	158 (9.59)	
Very frequently (>5 times/week)	28 (0.75)	10 (0.48)	18 (1.09)	
Daily screen time: 1–2 h	264 (7.05)	157 (7.49)	107 (6.50)	**0.018**
2–3 h	531 (14.19)	318 (15.17)	215 (13.05)	
3–4 h	879 (23.48)	511 (24.38)	361 (21.92)	
4–5 h	651 (17.39)	363 (17.32)	289 (17.55)	
5–6 h	505 (13.49)	261 (12.45)	241 (14.63)	
6 h or more	913 (24.39)	486 (23.19)	434 (26.35)	
Leisure-related physical activity: No	1,186 (31.69)	646 (30.82)	538 (32.67)	0.242
Yes	2,557 (68.31)	1,450 (69.18)	1,109 (67.33)	
Computer games-related physical activity: No	2,169 (57.95)	1,211 (57.78)	954 (57.92)	0.954
Yes	1,574 (42.05)	885 (42.22)	693 (42.08)	
Travel-related physical activity: No	2059 (55.01)	1,069 (51.00)	998 (60.60)	**< 0.001**
Yes	1,684 (44.99)	1,027 (49.00)	649 (39.40)	
Physical labor-related physical activity: No	2,390 (63.85)	1,347 (64.27)	1,047 (63.57)	0.685
Yes	1,353 (36.15)	749 (35.73)	600 (36.43)	
Mental labor-related physical activity: No	2,941 (78.57)	1,619 (77.24)	1,325 (80.45)	**0.019**
Yes	802 (21.43)	477 (22.76)	322 (19.55)	
Other physical activities: No	3,433 (91.72)	1917 (91.46)	1,520 (92.29)	0.391
Yes	310 (8.28)	179 (8.54)	127 (7.71)	
Daily natural light exposure: Almost never	177 (4.73)	71 (3.39)	106 (6.44)	**< 0.001**
< 30 min	801 (21.40)	444 (21.18)	357 (21.68)	
30 min to 1 h	1,523 (40.69)	862 (41.13)	661 (40.13)	
1–2 h	783 (20.92)	447 (21.33)	336 (20.40)	
> 2 h	459 (12.26)	272 (12.98)	187 (11.35)	
Siesta duration (min): 0	1,530 (40.88)	891 (42.51)	639 (38.80)	**0.024**
10	11 (0.29)	5 (0.24)	6 (0.36)	
15	27 (0.72)	14 (0.67)	13 (0.79)	
20	135 (3.61)	77 (3.67)	58 (3.52)	
30	598 (15.98)	324 (15.46)	274 (16.64)	
45	375 (10.02)	199 (9.49)	176 (10.69)	
60	754 (20.14)	438 (20.90)	316 (19.19)	
120	270 (7.21)	130 (6.20)	140 (8.50)	
180	43 (1.15)	18 (0.86)	25 (1.52)	
ALT (U/L)	24.82 ± 29.55	24.89 ± 27.65	24.72 ± 31.82	0.858
Creatinine (μmol/L)	66.47 ± 13.53	66.79 ± 13.59	66.06 ± 13.44	0.101
Uric acid (μmol/L)	374.99 ± 96.71	377.22 ± 95.45	372.14 ± 98.25	0.111
Urea (mmol/L)	4.72 ± 1.15	4.76 ± 1.14	4.68 ± 1.15	**0.037**
Red blood cells (×10^12^/L)	4.70 ± 0.49	4.71 ± 0.48	4.67 ± 0.50	**0.013**
Red cell distribution width (%)	13.15 ± 1.08	13.13 ± 1.06	13.18 ± 1.10	0.209
Red cell distribution width standard deviation (fL)	42.73 ± 2.36	42.7 ± 2.34	42.77 ± 2.37	0.387
Hemoglobin (g/L)	136.64 ± 16.8	137.3 ± 16.52	135.8 ± 17.12	**0.006**
Hematocrit (%)	42.42 ± 4.61	42.61 ± 4.55	42.17 ± 4.68	**0.004**
White blood cell count (×10^9^/L)	7.39 ± 1.91	7.36 ± 1.88	7.42 ± 1.95	0.381
Neutrophils (×10^9^/L)	4.54 ± 1.57	4.52 ± 1.54	4.57 ± 1.60	0.335
Neutrophil percentage (%)	60.59 ± 8.04	60.51 ± 8.09	60.68 ± 7.98	0.513
Monocytes (×10^9^/L)	0.47 ± 0.14	0.47 ± 0.14	0.47 ± 0.15	0.649
Eosinophils (×10^9^/L)	0.12 ± 0.12	0.12 ± 0.11	0.12 ± 0.13	0.510
Eosinophil percentage (%)	1.64 ± 1.56	1.66 ± 1.47	1.61 ± 1.67	0.262
Basophil percentage (%)	0.44 ± 0.24	0.44 ± 0.24	0.44 ± 0.24	0.898
Lymphocytes (×10^9^/L)	2.23 ± 0.61	2.22 ± 0.61	2.23 ± 0.61	0.606
Lymphocyte percentage (%)	30.87 ± 7.26	30.88 ± 7.32	30.85 ± 7.19	0.916
Platelets (×10^9^/L)	262.98 ± 58.65	261.65 ± 58.45	264.66 ± 58.88	0.119
PDW (%)	15.98 ± 0.38	15.99 ± 0.40	15.98 ± 0.35	0.452
Plateletcrit (%)	0.27 ± 0.05	0.27 ± 0.05	0.27 ± 0.05	0.498
Mean platelet volume (fL)	10.54 ± 1.22	10.57 ± 1.22	10.5 ± 1.22	0.063
Mean corpuscular volume (fL)	90.42 ± 4.86	90.49 ± 4.79	90.33 ± 4.96	0.333
Mean corpuscular hemoglobin (pg)	29.11 ± 1.98	29.14 ± 1.94	29.06 ± 2.03	0.242
Mean corpuscular hemoglobin concentration (g/L)	321.72 ± 9.86	321.87 ± 9.68	321.53 ± 10.09	0.292
BMI (kg/m^2^)	22.05 ± 4.08	22.1 ± 4.04	21.98 ± 4.12	0.350
SBP (mmHg)	116.09 ± 12.51	116.41 ± 12.85	115.69 ± 12.07	0.083
DBP (mmHg)	73.72 ± 7.86	73.66 ± 7.98	73.8 ± 7.72	0.609

*P* values less than 0.05 are given in bold.

PSQI scores stratified by individual characteristics are presented in Supplementary Table 2. Regarding lifestyle behaviors, students who rarely consumed caffeinated beverages had lower average PSQI scores (4.26 ± 2.42) than those who never consumed them (4.46 ± 2.71). However, no significant differences in PSQI scores were found across groups defined by daily screen time (both *p* > 0.05).

### 3.2 Association between sleep quality and biochemical indicators

The results of the MLR analysis are presented in Table 2. Male students exhibited a lower risk of poor sleep quality compared to female students (OR = 1.65; 95% CI: 1.32–2.06, *p* < 0.001). Similarly, undergraduate freshmen had a lower risk of poor sleep quality compared to junior college freshmen (OR = 0.53; 95% CI: 0.44–0.62, *p* < 0.001). In terms of lifestyle behaviors, longer daily screen time (OR = 1.08; 95% CI: 1.03–1.13, *p* = 0.001) and more frequent consumption of caffeinated beverages intake (OR = 1.11; 95% CI: 1.03–1.19, *p* = 0.003) had an increased risk of poor sleep quality. Students who engaging in travel-related physical activities (OR = 0.74; 95% CI: 0.63–0.85, *p* < 0.001) and not engaging in computer games-related physical activities (OR = 1.20; 95% CI: 1.03–1.39, *p* = 0.017) were more likely to have good sleep quality. Among biochemical indicators, higher serum urea levels were associated with a reduced risk of poor sleep quality, with each unit increase corresponding to a 13% reduction in the odds of poor sleep quality (OR = 0.87, 95% CI: 0.81–0.93, *p* < 0.001). Conversely, serum creatinine levels were associated with increased risk (OR = 1.01; 95% CI: 1.00–1.02, *p* = 0.002). A linear trend test further confirmed significant associations of urea levels (*p* = 0.016) and SBP (*p* = 0.018) with sleep quality.

**Table 2:** Risk factors for sleep quality identified by MLR.

Variable	OR	95% CI	z	*P*	*P* for trend
Age	0.94	0.84–1.05	−0.96	0.293	0.751^L^
Gender: Male				**< 0.001**	
Female	1.65	1.32–2.06	3.97		
College level: Junior college				**< 0.001**	
Undergraduate	0.53	0.44–0.62	−0.35		
Father educational level	0.98	0.84–1.12	−0.45	0.722	
Mother educational level	1.01	0.86–1.17	0.27	0.925	
Family economic status	0.74	0.60–0.88	−3.40	**0.001**	
Monthly living expense	0.67	0.56–0.79	−4.24	**< 0.001**	
Habitual caffeinated beverage intake	1.11	1.03–1.19	2.95	**0.003**	
Daily screen time	1.08	1.03–1.13	3.19	**0.001**	
Leisure-related physical activity: No				0.057	
Yes	0.85	0.72–1.00	−1.68		
Computer games-related physical activity: No				**0.017**	
Yes	1.20	1.03–1.39	2.38		
Travel-related physical activity: No				**< 0.001**	
Yes	0.74	0.63–0.85	−4.27		
Physical labor-related physical activity: No				0.157	
Yes	0.89	0.76–1.04	−1.54		
Mental labor-related physical activity: No				0.233	
Yes	0.90	0.75–1.07	−0.97		
Other physical activities: No				**0.007**	
Yes	0.70	0.53–0.90	−2.06		
Daily natural light exposure	0.94	0.87–1.00	−1.89	0.064	
Siesta duration	1.04	1.02–1.07	3.26	**< 0.001**	
ALT	1.00	0.99–1.00	1.80	0.317	0.532^L^
Creatinine	1.01	1.00–1.02	2.72	**0.002**	0.278^L^
Uric acid	1.00	0.99–1.00	0.18	0.681	0.615^L^
Urea	0.87	0.81–0.93	−3.55	**< 0.001**	**0.016** ^**L**^
Basophil percentage	1.00	0.74–1.33	−0.05	0.998	0.421^L^
PDW	0.90	0.72–1.11	−0.07	0.337	0.963^L^
BMI	0.99	0.96–1.01	−0.85	0.448	0.608^L^
SBP	1.01	0.99–1.02	−1.63	0.180	**0.018** ^**L**^
DBP	0.99	0.98–1.00	1.30	0.091	0.978^L^

*P* values less than 0.05 are given in bold. L stands for linear.

### 3.3 Mediating role of lifestyle in the sleep quality-renal function relationship

**Figure 2:**
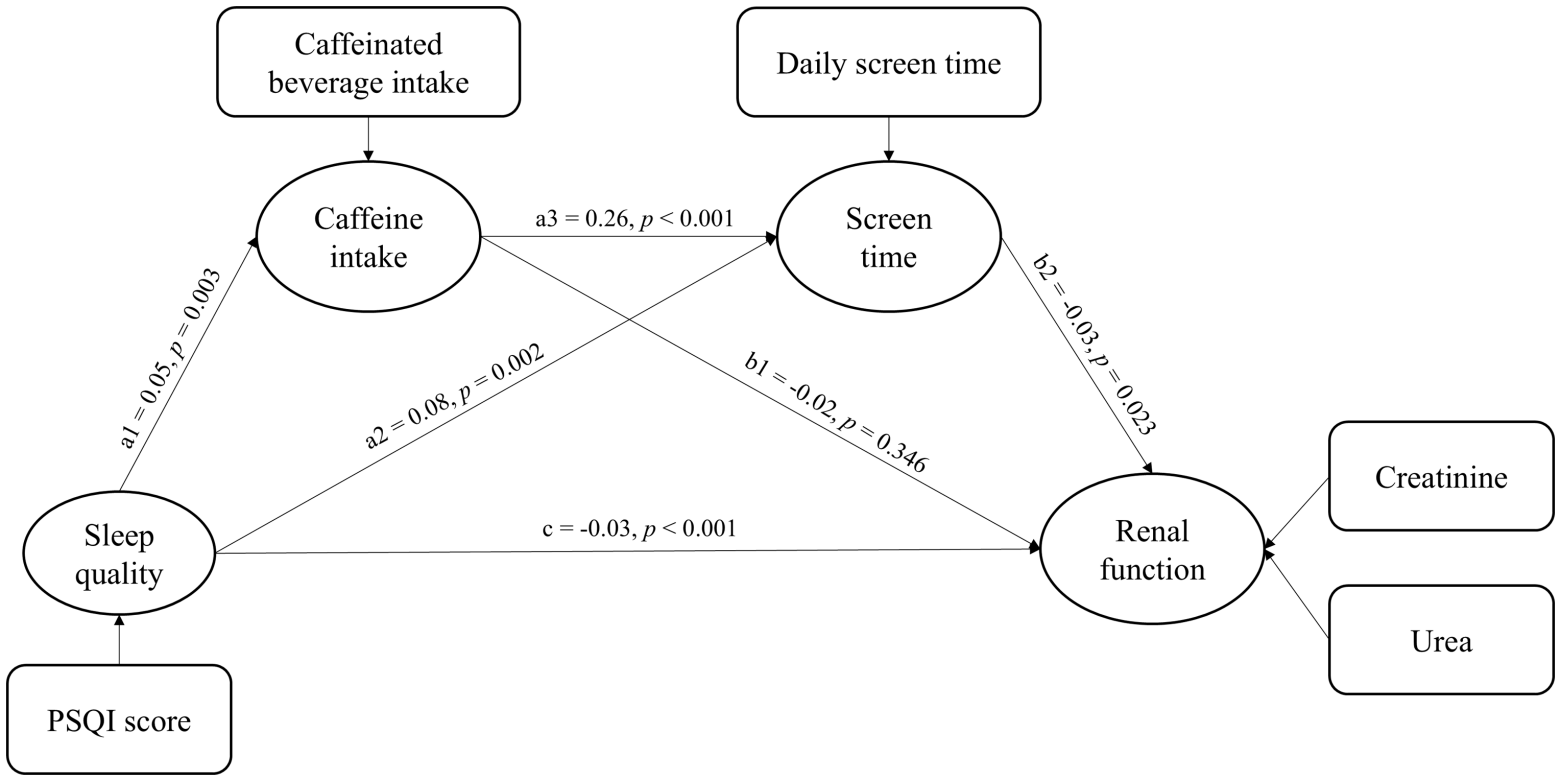
Median effect of life behaviors.

As illustrated in Figure 2, the model presents a chain mediation pathway linking sleep quality to renal function. The model demonstrates a good overall fit, as indicated by a CFI of 0.99 and a RMSEA of 0.02. Detailed path coefficients and their statistical significance are reported in Table 3. The total effect of sleep quality on renal function was not statistically significant (*β*: −0.01, *p* = 0.346), indirect effects via behavioral mediators were significant. Specifically, poor sleep quality was significantly associated with increased caffeinated beverage intake (*β* = 0.05, *p* = 0.003) and longer daily screen time (*β* = 0.05, *p* = 0.002). Daily screen time was negatively associated with renal function (*β* = −0.03, *p* = 0.023), whereas the effect of caffeinated beverage intake on renal function was not statistically significant (*β* = −0.01, *p* = 0.346). Additionally, caffeinated beverage intake was positively associated with daily screen time (*β* = 0.24, *p* < 0.001).

**Table 3:** Results of model path relationship hypothesis testing.

Pathway Relationship	Estimate	S. E.	95% CI		*p*
			Lower	Upper	
Sleep quality → Caffeine intake (a1)	0.05	0.02	0.00	0.08	**0.003**
Sleep quality → Daily screen time (a2)	0.05	0.02	0.03	0.11	**0.002**
Caffeine intake → Daily screen time (a3)	0.24	0.02	0.20	0.30	**< 0.001**
Caffeine intake → Renal function (b1)	−0.01	0.01	−0.04	0.00	0.346
Daily screen time → Renal function (b2)	−0.03	0.01	−0.10	−0.03	**0.023**
Sleep quality → Renal function (c)	−0.01	0.01	−0.07	−0.00	**0.346**

*P* values less than 0.05 are given in bold.

Table 4 showed the standardized estimates of each indirect path and the 95% CI of the mediating effect. The total direct effect of sleep quality on renal function was not statistically significant (*p* = 346). However, several indirect effects were observed. Specifically, the mediation effect value of daily screen time is −0.005 (*p* = 0.006). And the mediation effect value of chain mediation through caffeinated beverage intake and daily screen time is −0.001 (*p* = 0.010). These results reveal that caffeinated beverage intake and daily screen time partially mediate the effect of sleep quality on renal function through two pathways: (1) Sleep quality→daily screen time→renal function; (2) sleep quality→caffeinated beverage intake→daily screen time→renal function.

**Table 4:** Intermediate path relationship test results.

Trails	Standardization path factor	S. E.	95% CI		*p*
			Lower	Upper	
Sleep quality → Renal function	−0.012	0.013	−0.037	0.014	0.346
Sleep quality → Caffeine intake → Renal function	−0.002	0.001	−0.004	0.000	0.084
Sleep quality → Daily screen time → Renal function	−0.005	0.002	−0.009	−0.002	**0.006**
Sleep quality → Caffeine intake →Daily screen time → Renal function	−0.001	0.001	−0.002	0.000	**0.010**

*P* values less than 0.05 are given in bold.

### 3.4 Stratified analysis by gender

Among female students, sleep quality was significantly associated with both creatinine and urea levels. Higher creatinine levels had an increased risk of poor sleep quality (OR = 1.01; 95% CI: 1.00–1.03, *p* = 0.005), whereas higher urea levels were associated with reduced risk (OR = 0.84; 95% CI: 0.76–0.92, *p* < 0.001). In male students, the inverse association between urea levels and poor sleep quality remained significant (OR = 0.90; 95% CI: 0.81–0.99, *p* = 0.048), while the association with creatinine was not statistically significant (OR = 1.01; 95% CI: 0.99–1.01, *p* = 0.152). Notably, higher SBP (OR = 0.98; 95% CI: 0.97–0.99, *p* = 0.002) was associated with a decreased risk of poor sleep quality among males (Table 5).

**Table 5:** Stratified analyses of the association between sleep quality and biochemical indicators by gender.

Variables	Male			Female		
	OR	95% CI	*P*	OR	95% CI	*p*
ALT	1.00	1.00–1.00	0.069	1.00	0.99–1.01	0.588
Creatinine	1.01	0.99–1.01	0.152	1.01	1.00–1.03	**0.005**
Uric acid	1.00	0.99–1.00	0.991	1.00	0.99–1.00	1.000
Urea	0.90	0.81–0.99	**0.048**	0.84	0.76–0.92	**< 0.001**
Basophil percentage	1.12	0.75–1.65	0.583	0.86	0.56–1.31	0.483
PDW	0.79	0.56–1.08	0.148	1.00	0.73–1.34	0.975
BMI	1.02	0.98–1.04	0.334	0.97	0.93–1.00	0.083
SBP	0.98	0.97–0.99	**0.002**	1.00	0.98–1.01	0.854
DBP	1.01	0.99–1.02	0.303	1.01	0.99–1.02	0.214

*P* values less than 0.05 are given in bold.

## 4 Discussion

This study investigated the associations between sleep quality and renal function indicators (serum creatinine and urea level) among incoming college freshmen from Jiangsu, China. Higher creatinine levels were positively associated with poor sleep quality, whereas higher urea levels were inversely associated. These associations were more pronounced among female participants. Additionally, SEM analysis demonstrated that caffeine beverage intake and daily screen time partially mediated the relationship between sleep quality and renal function. These findings underscore potential behavioral pathways for targeted health interventions in college populations.

In this study, the association between urea levels and sleep quality may reflect more effective nitrogen metabolism during sleep. One plausible mechanism involves the circadian regulation of ornithine carbamoyltransferase, a key hepatic enzyme in the urea cycle. The expression of ornithine carbamoyltransferase shows diurnal oscillations and is sensitive to sleep disruption, suggesting that adequate sleep may help maintain the rhythmic activity of the urea cycle and promote efficient ammonia detoxification (15). Supporting evidence from metabolomic studies in athletic populations has shown that improved sleep efficiency is linked to elevated levels of urea cycle and Krebs cycle metabolites following intense exercise, highlighting the role of sleep in metabolic recovery (16). In addition, studies in diabetic nephropathy models suggest that melatonin, a hormone influenced by sleep, improves renal function by reducing blood urea nitrogen (BUN) levels. This effect may be mediated through upregulation of Beclin1 expression in mesenchymal stem cells, enhancing autophagy and providing renal protection (17). Contrasting results have been observed in clinical populations, particularly among patients with diabetes or chronic kidney dysfunction, where elevated BUN levels are often regarded as markers of metabolic burden, and have been linked to poorer sleep outcomes (18). This discrepancy does not conflict with our findings, as our study focused on a healthy population with urea levels within the normal physiological range (a mean urea concentration of 4.72 mmol/L in this study corresponds to a BUN of 2.21 mmol/L) (19). The observed association likely reflects non-pathological metabolic variations influenced more by nutritional status and lifestyle choices than by renal impairment. These differences in population health status and baseline renal function may help explain the inconsistencies across studies.

Creatinine levels exhibited a positive correlation with poor sleep quality in this study. This finding is supported by Mendelian randomization results, which demonstrated causal relationships: insomnia was associated with elevated serum creatinine levels (OR = 1.04, 95% CI: 1.00–1.08), while short sleep duration was inversely associated (OR = 0.96, 95% CI: 0.93–0.98) with serum creatinine levels (20). These observations are consistent with previous research conducted in populations with chronic kidney disease (21). Several pathophysiological mechanisms may underlie the association. Poor sleep quality can activate the sympathetic nervous system and blunt nocturnal blood pressure dipping, adversely affecting renal hemodynamics. Second, intermittent hypoxia, common in sleep disorders such as obstructive sleep apnea, can induce oxidative stress and systemic inflammation, contributing to endothelial dysfunction within the kidneys. Moreover, poor sleep quality is closely linked to an increased risk of hypertension, diabetes, and obesity, all of which are well-established risk factors for chronic kidney disease and may indirectly impair renal function (18, 22).

Notably, in our subgroup analysis, the positive association between sleep quality and serum creatinine levels was more pronounced among females. This sex-specific vulnerability is consistent with findings from a study in Korean women, which reported a positive link between longer sleep duration and higher serum creatinine levels. Both physiological and psychosocial factors may contribute to this disparity. Physiologically, approximately one-third of women experience sleep disturbances and related symptoms, such as cramps, bloating, and headaches, during the premenstrual period or menstruation. Psychosocially, women have a higher prevalence of depression beginning in adolescence, which is closely linked to insomnia and altered sleep patterns (23). These factors may collectively exacerbate the impact of sleep disturbances on renal function in women.

Lifestyle factors may also contribute to the observed associations between sleep quality and renal function. The present study shows that the prevalence of poor sleep quality among incoming college freshmen is 44.05%, exceeds the rates reported at other studies conducted in China (24–26). Unlike the structured routine of school days, less regulated periods such as holidays may foster unhealthy lifestyle behaviors that adversely impact sleep quality (27). During this period, more than half of the participants reported consuming caffeinated beverages at least once per week, a trend often driven by social interactions, emotional regulation, and fatigue management (28). Caffeine as a well-established and prevalent risk factor for poor sleep quality, delays sleep onset and reduces sleep depth (29). It is frequently accompanied by prolonged screen exposure, especially at night, perpetuating poor sleep hygiene. Blue light from screens can delay sleep onset, suppress melatonin, and disrupt circadian rhythms, with potential effects on renal function. Excessive screen time has also been linked to neurostructural alterations that may indirectly affect both sleep and kidney health (21, 30).

Interestingly, our stratified analysis revealed higher SBP level had a lower risk of poor quality in males, which contrast with conventional research findings. This unexpected result may reflect the unique physiological characteristics of young males, such as elevated basal sympathetic activity and less stable blood pressure regulation, which can persist even in the presence of good sleep quality (31). Importantly, all SBP values in our sample remained within the normal range, suggesting that these variations are likely physiological rather than pathological. It is also possible that individuals with slightly elevated SBP are more health-conscious and thus adopt compensatory behaviors that support better sleep. Nevertheless, residual confounding from factors such as stress, diet, and physical activity cannot be excluded. Future studies employing objective sleep measures are needed to further clarify these associations.

This study offers several novel contributions. First, this study identified early renal biochemical indicators alterations in ostensibly healthy college freshmen with poor sleep quality. The inclusion of both undergraduate and junior college freshmen (primarily from Jiangsu province) enhances the generalizability of our findings, supporting targeted health interventions. Secondly, by incorporating renal function indicators (creatinine and urea) into the SEM, we provide a novel approach to assessing early renal risk from an integrative perspective. Thirdly, the study elucidates a sequential mediation pathway involving caffeine intake and daily screen time, thereby expand the lifestyle-related mechanisms linking sleep and renal health. Finally, the application of multiple rigorous statistical methods, including regression diagnostics, strengthens the reliability of our results.

However, several limitations must be noted. At first, the cross-sectional design limits causal inference, and the temporal sequence between sleep quality and renal function cannot be firmly established. Second, in consideration of the questionnaire length, this study did not include comprehensive measures for potential confounders such as dietary patterns, psychological health, and academic stress. Thirdly, the PSQI used in this study assesses overall sleep quality, but does not capture specific sleep disturbances. Future research could explore more detailed sleep issues to better understand the impact on renal function. Fourth, the reliance on self-reported lifestyle behaviors may have introduced measurement error. Longitudinal studies using objective measures are needed to validate and extend our findings. Finally, as the sample was limited to Chinese college freshmen, the generalizability of the results to other populations may be constrained.

## 5 Conclusion

In this relatively healthy population of Chinese incoming college freshmen, we identified a significant association between sleep quality and renal function indicators, with more pronounced effect observed among female students. These findings underscore the importance of early attention to renal health, even in young and ostensibly healthy populations. The sex-specific differences observed suggest that female students may be more vulnerable to the effects of poor sleep and renal function. Moreover, the mediating role of lifestyle factors, such as caffeine consumption and screen time, highlights the potential of targeting modifiable behaviors to improve sleep quality and renal health. These findings provide practical implications for early interventions aimed at promoting healthy sleep and lifestyle habits to protect renal function in college freshmen. Nonetheless, the cross-sectional design of this study limits causal inference. Future longitudinal studies incorporating objective sleep assessments and clinical renal outcomes are warranted to validate and extend these findings.

## Data Availability

The original contributions presented in the study are publicly available. This data can be found here: https://gitee.com/xiaostat/sleep-qualityand-renal-function.

## Footnotes

^1^ www.wjx.cn
