# Supplementary material for: Sleep quality and renal function among Chinese incoming college freshmen: the mediating role of lifestyle behaviors
Source: Front Public Health. 2025 Jun 6;13:1502947. doi: 10.3389/fpubh.2025.1502947 (PMC12179153; doi:10.3389/fpubh.2025.1502947)
Supplement: Supplementary file 1 [file Data_Sheet_1.docx]

Supplementary Material

# Supplementary Figures and Tables

## Supplementary Figures


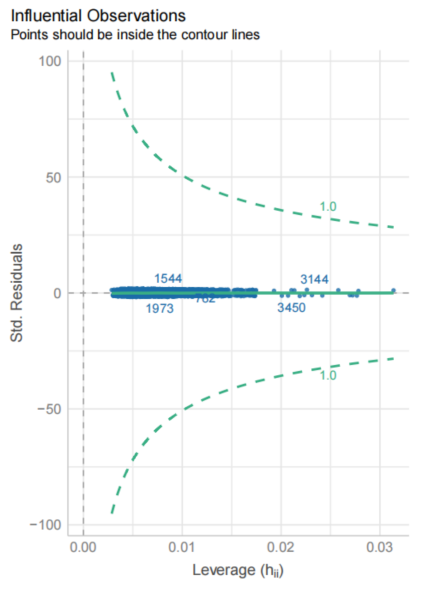


**Supplementary Figure 1.** Diagnostics for extreme values in MLR performance


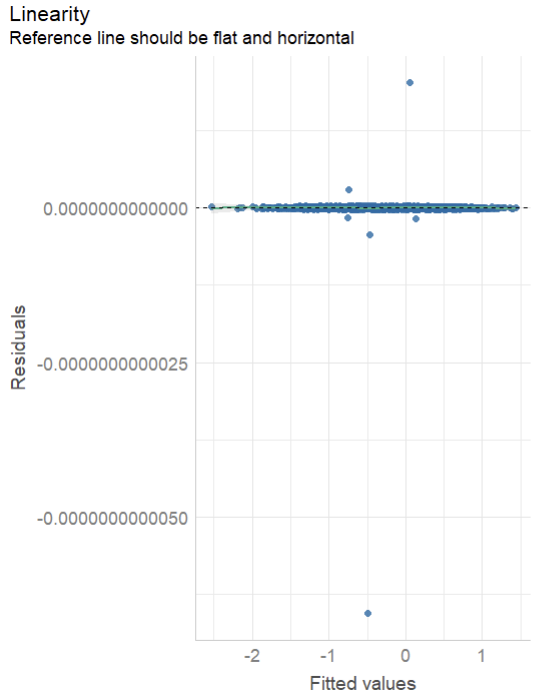


**Supplementary Figure 2.** Diagnostics for linearity in MLR performance


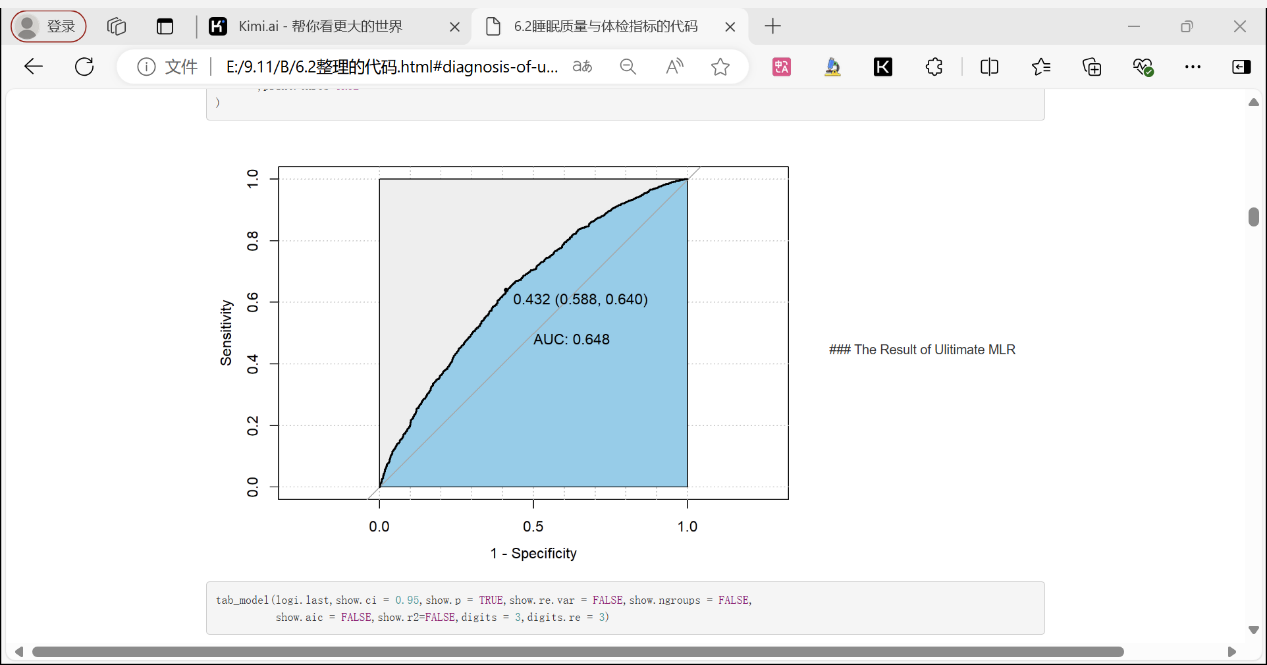


**Supplementary Figure 3.** ROC curve of the MLR


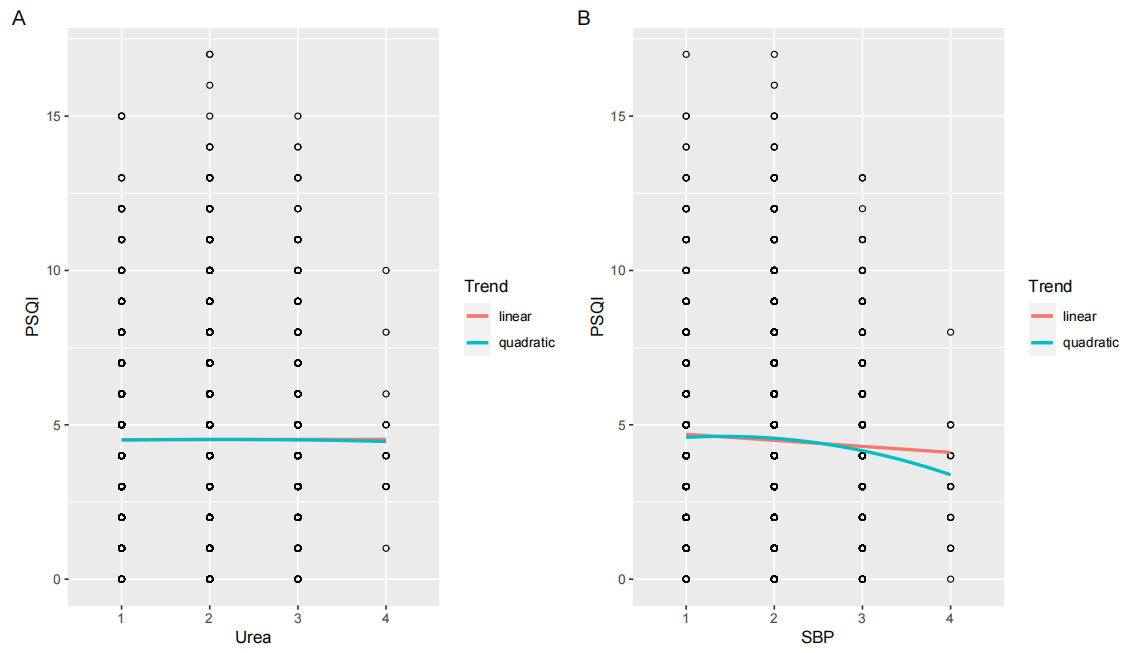


**Supplementary Figure 4.** Linear trend between urea, SBP, and sleep quality in MLR

## Supplementary Tables

**Supplementary Table 1.** The geographic distribution of participants across provinces

| Province | n (%) |
| --- | --- |
| Anhui | 125 (3.34) |
| Chongqing | 4 (0.11) |
| Fujian | 12 (0.32) |
| Gansu | 12 (0.32) |
| Guangdong | 7 (0.19) |
| Guangxi Zhuang ethics | 19 (0.51) |
| Guizhou | 14 (0.37) |
| Hainan | 6 (0.16) |
| Hebei | 30 (0.80) |
| Heilongjiang | 3 (0.08) |
| Henan | 107 (2.86) |
| Hubei | 13 (0.35) |
| Hunan | 19 (0.51) |
| Inner Mongolia | 7 (0.19) |
| Jiangsu | 3044 (81.33) |
| Jiangxi | 14 (0.37) |
| Jilin | 7 (0.19) |
| Liaoning | 6 (0.16) |
| Ningxia | 6 (0.16) |
| Qinghai | 9 (0.24) |
| Shandong | 105 (2.81) |
| Shanghai | 4 (0.11) |
| Shanxi | 51 (1.36) |
| Shanxi1 | 9 (0.24) |
| Sichuan | 24 (0.64) |
| Tianjin | 1 (0.03) |
| Xinjiang Uyghur | 42 (1.12) |
| Xizang | 13 (0.35) |
| Yunnan | 11 (0.29) |
| Zhejiang | 19 (0.51) |

**Supplementary Table 2.** PSQI scores of participants with different characteristics

| Variables | n | Percent | PSQI | *P* |
| --- | --- | --- | --- | --- |
| Age (year) |  |  |  |  |
| Gender: Male | 1741 | 46.51 | 4.40±2.64 |  |
| Female | 2002 | 53.49 | 4.64±2.50 |  |
| College level: Junior college | 2374 | 63.43 | 4.83±2.70 |  |
| Undergraduate | 1369 | 36.57 | 4.00±2.25 |  |
| Father educational level: High school or below | 2811 | 75.10 | 4.62±2.56 | a |
| Junior college | 521 | 13.92 | 4.39±2.65 | a |
| College or higher | 411 | 10.98 | 4.07±2.51 | b |
| Mother educational level: High school or below | 2960 | 79.08 | 4.63±2.57 | a |
| Junior college | 478 | 12.77 | 4.18±2.49 | b |
| College or higher | 305 | 8.15 | 4.07±2.59 | b |
| Family economic level: Poor | 484 | 12.93 | 5.29±2.70 | a |
| Fair | 3111 | 83.12 | 4.44±2.54 | b |
| Good | 148 | 3.95 | 3.86±2.31 | c |
| Monthly living expense: < 1000 | 633 | 16.91 | 5.24±2.82 | a |
| 1000-2000 | 2872 | 76.73 | 4.43±2.50 | b |
| ＞2000 | 238 | 6.36 | 3.82±2.36 | c |
| Habitual caffeinated beverage intake: Never | 1036 | 27.68 | 4.46±2.71 | a |
| Rarely (1-2 times/month) | 650 | 17.37 | 4.26±2.42 | a, b |
| Occasionally (1-2 times/week) | 1702 | 45.47 | 4.59±2.50 | b |
| Frequently (3-5 times/week) | 327 | 8.74 | 4.87±2.68 | b, c |
| Very frequently (>5 times/week) | 28 | 0.75 | 5.50±3.16 | c |
| Daily screen time: 1-2 hours | 264 | 7.05 | 4.21±2.75 | a |
| 2-3 hours | 533 | 14.24 | 4.38±2.65 | a |
| 3-4 hours | 872 | 23.30 | 4.46±2.53 | a |
| 4-5 hours | 652 | 17.42 | 4.47±2.54 | a |
| 5-6 hours | 502 | 13.41 | 4.67±2.47 | a |
| 6 hours or more | 920 | 24.58 | 4.74±2.57 | a |
| Leisure-related physical activity: No | 1184 | 31.63 | 4.59±2.63 |  |
| Yes | 2559 | 68.37 | 4.5±2.54 |  |
| Computer games-related physical activity: No | 2165 | 57.84 | 4.53±2.62 |  |
| Yes | 1578 | 42.16 | 4.52±2.50 |  |
| Travel-related physical activity: No | 2067 | 55.22 | 4.73±2.61 |  |
| Yes | 1676 | 44.78 | 4.27±2.50 |  |
| Physical labor-related physical activity: No | 2394 | 63.96 | 4.51±2.57 |  |
| Yes | 1349 | 36.04 | 4.55±2.58 |  |
| Mental labor-related physical activity: No | 2944 | 78.65 | 4.59±2.58 |  |
| Yes | 799 | 21.35 | 4.29±2.52 |  |
| Other physical activities: No | 3437 | 91.82 | 4.54±2.61 |  |
| Yes | 306 | 8.18 | 4.34±2.15 |  |
| Daily natural light exposure: Almost never | 177 | 4.73 | 5.60±3.18 | a |
| < 30 min | 801 | 21.40 | 4.67±2.58 | b |
| 30 min to 1 hour | 1523 | 40.69 | 4.47±2.56 | b |
| 1-2 hours | 783 | 20.92 | 4.35±2.27 | b |
| > 2 hours | 459 | 12.26 | 4.34±2.72 | b |
| Siesta duration: 0 min | 1530 | 40.88 | 4.45±2.50 | a |
| 10 min | 11 | 0.29 | 6.27±4.15 | a |
| 15 min | 27 | 0.72 | 5.04±3.04 | a |
| 20 min | 135 | 3.61 | 4.59±2.61 | a |
| 30 min | 598 | 15.98 | 4.59±2.67 | a |
| 45 min | 375 | 10.02 | 4.75±2.79 | a |
| 60 min | 754 | 20.14 | 4.38±2.48 | a |
| 120 min | 270 | 7.21 | 4.61±2.36 | a |
| 180 min | 43 | 1.15 | 5.49±3.13 | a |
